# Humidifying, heating and trap-density effects on triple-cation perovskite solar cells

**DOI:** 10.1038/s41598-023-40837-8

**Published:** 2023-08-18

**Authors:** Leila Yadegari, Zahra Rastegar Moghadamgohari, Nazila Zarabinia, Reza Rasuli

**Affiliations:** Department of Physics, Faculty of Science, University of Zanjan, P.O. Box 45371-38791, Zanjan, Iran

**Keywords:** Solar cells, Solar cells

## Abstract

The effect of moisture and heat are important challenges in perovskite solar cells (PSCs). Herein we studied the performance of triple-cation PSCs in different operating environmental conditions. Humidified cells exhibited a hopeful character by increasing the open-circuit voltage (*V*_*OC*_) and short-circuit current density (*J*_*SC*_) to 940 mV and 22.85 mA cm^−2^ with a power conversion efficiency (PCE) of 14.34%. In addition, further analyses showed that hysteresis index and charge transfer resistance decrease down to 0.4% and 1.67 kΩ. The origin of superior stability is ion segregation to the interface, which removes the antisite defect states. Finally, the effect of operating temperature and trap density on structure and performance was also studied systematically.

## Introduction

Perovskite solar cells (PSCs) have been considered a promising device due to the low-cost elemental components and high-power conversion efficiency (PCE)^1–4^. The efficiency of the stable PSCs has enhanced from 0.66% in 1996 to 25.2% at present^2,5^. There is a large difference between the PCE of the most durable and the high-efficiency PSCs, and stability is the primary privilege of PSCs^6,7^. As known, full coverage of the perovskite film is necessary for sufficient shunt resistance, and plenty of atomic disorder in perovskite during the deposition process causes lower *V*_*OC*_ and *J*_*SC*_^8^.

To obtain an efficient perovskite layer, deposition conditions and post-processing treatment were extensively studied on the perovskite layer’s physical properties^9–11^. Although these processes can reduce structural defects and electronic traps, however, perovskites typically decompose and de-wet at temperatures above 110 °C^12,13^. The diffusion length of charge carriers limits the perovskite film thickness, and photovoltaic performance decreases for thicker layers^14,15^. However, a hot substrate for layer deposition improves photovoltaic performance^16^, and separate crystals of perovskite can be fused by post-annealing or increasing the film thickness^12^.

On the other hand, moisture in the structure of perovskite is known as a suffering issue to the efficient performance of PSCs^17,18^. The quality of perovskite layer depends on the deposition process and atmospheric condition during the crystal growth^19^. Controlling the relative humidity of perovskite surface^20^ can tune the quality of grain boundaries in the perovskite layer due to crystallinity effects. As reported, the crystal quality is improved due to intermediate hydrate perovskite phase, which is formed during the annealing process^21^. Accordingly, many researchers have tried to use proper precursors to fabricate cells in atmospheric conditions. Using lead thiocyanate precursors, high-quality perovskite films can be prepared under humidity conditions^22^. As known, third cation substitution (such as Cs) in double cation perovskite of FA/MA can increase the formation energy of defects with a favorable Goldschmidt tolerance factor and adjust the absorber bandgap to match the buffer layer^23^. Triple-cation PSCs with Cs_0.05_(FA_0.83_MA_0.17_)_0.95_Pb(I_0.83_Br_0.17_)_3_ perovskite formula was noted as an optimized composition with good stability^24–26^. As reported previously, environmental stability is promising in triple-cation PSCs due to different cations in lead-halide perovskite materials^6,27,28^.

In this letter, we investigated the effect of moisture and heating on grain-growth perovskite films in triple-cation PSCs. The triple-cation PSCs show superior photovoltaic performance in presence of moisture due to grain-growth of perovskite film. Exposing perovskite films to moisture in a humid environment increases the grain size. Results show lower defects in triple-cation PSCs after humidifying, which increase charge carrier lifetime and reduce non-radiative recombination.

## Materials and methods

The used materials from Merk company are Titanium (IV) isopropoxide (TTIP, 97%), HCl (99%), anhydrous ethanol (99.5%), *N*, *N*-dimethylformamide (DMF, 99.8%), cesium iodide (CsI, 99.99%), dimethyl sulfoxide (DMSO, 99.9%). In addition, fluorine—doped tin oxide (FTO), lead (II) bromide (PbBr_2_, 99%), formamidinium iodide (FA, 98%), PbI_2_, methylammonium bromide (MA, 98%), CuInS, Spairo-OMeTAD materials were purchased from IRASOL.

To fabricate solar cells, FTO substrates were etched by drop-casting the 2 M HCl on zinc powders covered region of FTO and then cleaned by DI water, ethanol, isopropanol, and acetone. The prepared substrates were annealed at 500 °C for 1 h. To deposit compact TiO_2_, we solved TTIP in ethanol (0.24 M) and then coated it using a spin coater at 2000 rpm for 30 s. Afterward; the prepared samples were heated at 500 °C for 1 h. In the next step, a TiO_2_ paste was prepared in ethanol (1:5.5), and then deposited on the compact layer using a spin coater with an angular velocity of 4000 rpm for 30 s and then heated at 100 °C for 10 min. To obtain a mesoporous TiO_2_ scaffold, the prepared samples were annealed at 500 °C for 1 h. Perovskite solution was prepared by dissolving PbI_2_ (1.5M) and PbBr_2_ (1.5M) precursors in the mixture of DMF: DMSO (4:1), and then heated at 150 °C for 10 min. Afterward, MA and FA precursors were dissolved in PbBr_2_ and PbI_2_ solutions, separately, followed by adding 0.0015 mol CsI solution in 1 mL DMSO. The prepared perovskite solutions were dropped on the mesoporous TiO_2_ using a spin coater at 1000 and 4000 rpm in a glove box for 10 and 20 s, respectively. Then, the substrates were heated on the hotplate at 100 °C for 1 h. Finally, the copper indium sulfide (CIS) and Au contact was deposited using the thermal evaporation method.

The prepared cells were studied by an X-ray diffraction diffractometer (XRD) using a D8 ADVANCE apparatus from Bruker by Cu–k_α_ and a scanning electron microscope (SEM) using a TESCAN microscope. An AvaSpec-125 spectrophotometer from Avantes Company was utilized to measure the photoluminescence (PL) spectra using a 405 nm laser, illuminated to the front face of the prepared cells. The photovoltaic performance was measured using an IRASOL SIM-1000 solar simulator system under visible light illumination (AM 1.5 G, 100 mW cm^−2^). The electrochemical impedance spectroscopy (EIS) was performed using an Electrochemical Workstation ZIVE with ZMAN software in the dark in the frequency range of 1MHz to 0.01 Hz. The incident photon to electron conversion efficiency (IPCE) spectrum was calculated by IPCE-018 IRASOL instrument.

To perform a humidity test, the prepared solar cells were placed in a humidity chamber for 30 min that had been prepared at 55% relative humidity for 2 h. In addition, to investigate the heating effects, the prepared samples were heated at 80 °C for 1 h.

## Results and discussion

### Solar cell performance

**Figure 1:**
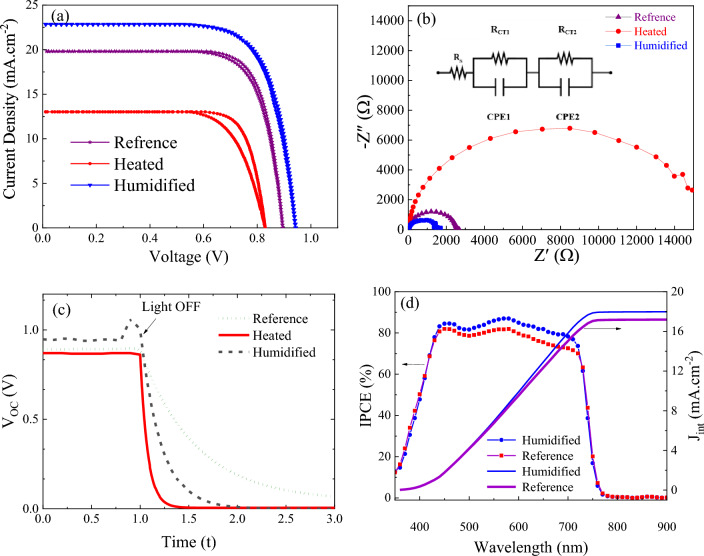
(**a**) J–V diagram of the cells after heating and humidifying. The results of the measurements are reported in Table 1, (**b**) the Nyquist diagram for the heated, humidified, and reference samples. These spectra were measured in the frequency range of 0.01 Hz. (**c**) Voltage decay of cells after humidifying and heating, (**d**) the IPCE and integrated current of the humidified and reference cells.

Figure 1 and Table 1, show the photovoltaic characteristics of the tested PSCs. We fabricated the devices with structure flexible-glass/ITO/SnO_2_/triple-cation perovskite/CIS/Au with a 0.16 cm^2^ active area. As presented, the reference cell with triple-cation perovskite shows PCE of 12.92%, *J*_*SC*_ of 19.81 mA cm^−2^, *V*_*OC*_ of 890 mV, and *FF* of 77%. However, the humidified cell shows higher PCE, *J*_*SC*_*, V*_*OC*_, and *FF* with a value of 14.34%, 22.85 mA cm^−2^, 940 mV, and 72%, respectively. To study the recombination dynamics and internal resistances of the PSCs, we conducted an EIS experiment. Figure 1b shows the results of EIS from the treated cells with humidity and heat, and the equivalent circuit is shown as an inset. The Nyquist plot of EIS for PSCs typically shows two semicircles due to the charge recombination at low frequencies and the Au counter electrode at high frequencies^29^. In our study, we observed just the low-frequency semicircle. According to the equivalent circuit in the inset of Fig. 1b, we fitted the experimental data. Table 1 presents the charge transfer resistance (*R*_*CT*_), the series resistance of the cells (*R*_*s*_), chemical capacitance (*C*_*μ*_), and electron lifetime (*τ*). As shown in Table 1, the humidified cell exhibits the lowest *R*_*CT*_, which facilitates charge transfer and increases the photocurrent. The size of the semicircles enhances after heat treatment by reaching a maximum of 14.36 kΩ, showing higher charge transfer resistance that decreases the cell performance. However, we know that slower recombination leads to a larger diameter of the EIS semicircle^30^. This can be due to Cs cation segregation to the interfaces that can induce a decrease in the charge transfer resistance.

**Table 1:** Photovoltaic parameters of the fabricated solar cells and electric properties of the fitted circuit for EIS results.

Sample	*V*_*OC*_ (mV)	*J*_*SC*_ (mA cm^−2^)	*FF* (%)	*η* (%)	HI (%)	*R*_*s*_ (Ω)	*R*_*CT*_ (kΩ)	*C*_*µ*_ (nF)	*τ* (µs)
Reference	890	19.81	77	12.92	2.97	9.14	2.43	8.60	20.89
Heated	830	13.01	79	8.13	9.35	15.45	14.36	9.05	130.18
Humidified	940	22.85	72	14.34	0.40	10.17	1.67	10.17	9.06

Forward and reverse J–V curve measurements in Fig. 1a show hysteresis in the prepared PSCs. The hysteresis index is determined according to the following formula:

1 $$ HI = \frac{{PCE_{r} - PCE_{f} }}{{PCE_{r} }} $$

The backward or reverse scan direction in the J–V measurement is done by the bias voltage sweeping from positive to negative (i. e., open-circuit to short-circuit); in contrast, the forward scan is in the opposite direction. Typically, the origin of hysteresis is ion migration, ferroelectricity, charge trapping, and capacitive effects. Hysteresis is a negative feature in solar cells, so scientists are always looking for its origin to reduce or eliminate it. To measure the exact extent of hysteresis, we must gain a proper understanding of its origin. Hysteresis consists of two types, normal and inverse, which are observed in the J–V measurement. Hysteresis is due to the device’s structure, the slow motion of the ions, the recombination of the electrons and holes, the ion migration, the capacitive effect, charge trapping, the scanning rate, and the environmental conditions. The observed hysteresis may also be a combination of these factors, although the exact origin of hysteresis in perovskite solar cells is still unknown. As shown in Fig. 1a, the heated samples’ hysteresis is 9.35% greater than the humidified cells (0.40%). This indicates that ion migration and charge trapping, and capacitive effects reduce after humidifying.

Figure 1c shows the *V*_*OC*_ decay for the heated, humidified, and reference PSCs. In Table 1, the electron lifetime (τ) was determined from^31^:

2 $$ \tau = - \frac{{k_{B} T}}{e}\left( {\frac{{dV_{OC} }}{dt}} \right)^{ - 1} $$

where *τ* is the electron lifetime, and *t* is the time. In addition, ${k}_{B}$ and $T$ represent the Boltzmann constant and test temperature, respectively. In Eq. (2), As shown in Table 1, electron lifetime increases from 20.89 to 130.18 µs for heated cells while humidity lowers *τ* down to 9.06 µs. Electron lifetime is dependent on Shunt current and radiative or non-radiative recombination. According to EIS and PL results, *R*_*CT*_ and *R*_*s*_ decrease for humidified cells probably due to Cs cation segregation in the interface, and this causes to decreasing electron lifetime. Figure 1d shows the IPCE of prepared cells before and after humidification. The IPCE is defined as the ratio of output electrons to the incident photons at different wavelengths. As shown, humidified cells exhibit a broad band with IPCE value greater than 80% in the wavelength range of 350–900 nm. It can be attributed to the Fabry–Perot resonances effects with efficient coupling into the waveguide modes of the absorber layer^32^. Furthermore, the integrated current density (J_int_) is 17.19 mA cm^−2^ that is increased after humidification to 17.98 mA cm−^-2^. The IPCE were measured without white light bias and its setup uses a Xe lamp with a monochromator as light source and consequently the light intensity is lower than the standard 1 sun illumination.

**Figure 2:**
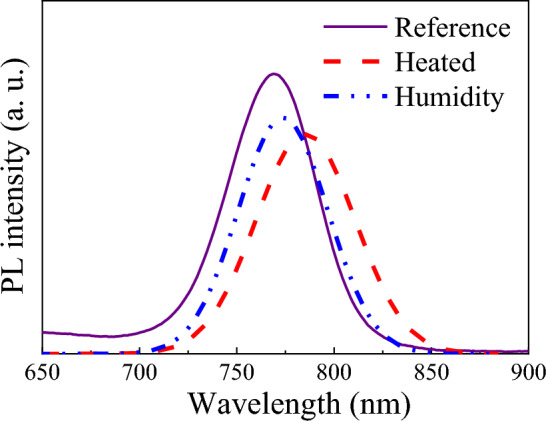
PL spectra from the reference, humidified, and heated samples.

According to the PL results, the peak intensity decreases for the humidified cell. Therefore, improving cell performance can be attributed to the decrease in radiative recombination, which is lower than that of the reference cell. When water molecule has a barrier to influence perovskite with negligible effect on charge localization, polar vibrations of water molecules increase nonadiabatic coupling and increase recombination. Therefore, radiative recombination decreases for humidified cells^33^. However, according to the heated cell data, a decrease in photovoltaic parameters besides the similar behavior in the PL peak suggests that the cell resistance increases due to heating the cell. From PL analysis in Fig. 2, the absorption edge shows a blue shift for the heated cell, reflected in the *J*_*SC*_. However, a humidified cell shows lower bandgap energies and does not suffer optical absorption and recombination rates than the references. For the heated PSC, the ammonium-based spacer limits the halide octahedral arranging in two dimensions and therefore decreases PCE. Besides, dielectric confinement in perovskites also influences their performance in the PSCs^29^.

As shown in Fig. 2, the cell’s corresponding PL peak position shows a shift after humidifying and heating with a value of 4.2 and 13.7 nm. The PL intensity varies due to the crystalline perovskite structures, confining the trap states formation and decreasing the defect density in the absorber layer. Hence, one can conclude that light absorption should be decreased in the heated cell while the humidified cell shows no considerable change in harvesting ability than the reference sample.

**Figure 3:**
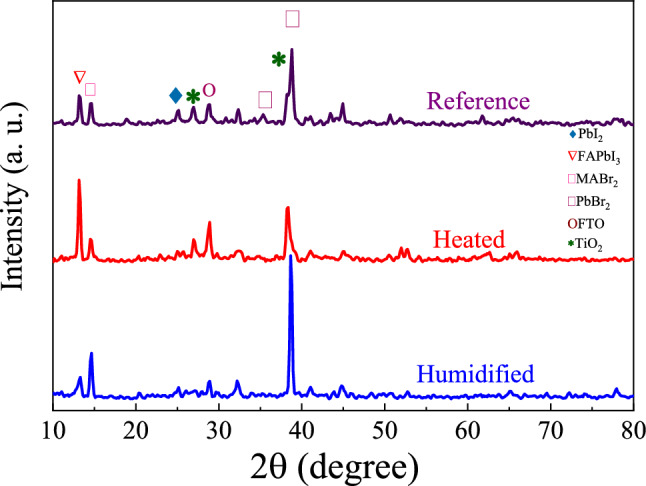
XRD patterns of reference, humidified, and heated PSCs.

The XRD patterns of the prepared PSCs after humidifying and heating are presented in Fig. 3. Specified peaks at the XRD pattern in Fig. 3 show that the cell layers have been crystallized as well^34^. The diffraction peaks at 14.5°, 19.2°, and 28.8° are assigning to the (1 1 0), (2 0 0), and (2 2 0) crystal planes of tetragonal perovskite^34^. The reference cell show peaks at 2θ = 13.3° and 25.0°, which are characteristic peaks of PbI_2_^35^. Although the PbI_2_ peaks were decreased with humidifying and heating, the peak at 2θ = 12.5° is observable for all samples, showing a negligible change in the structure of PbI_2_. Since XRD results do not provide information about non-stoichiometric species, this can be attributed to the diffusion of ions to the grain boundaries or interfaces. To investigate the grain size of perovskites, we calculated crystallite size by Scherrer’s relation^36^. According to XRD results, the perovskite crystallite size increases gradually from 12.6, 21.1 and 12.6 nm up to 64.0, 32.0 and 33.7 nm for PbBr_2_, MABr_2_ and FAPbI_3_, respectively. It can be concluded that grain size of perovskite increases accordingly as crystallite size increases. However, humidity destroys the crystal structure of PbI_2_, which segregates to the grain boundaries and interfaces that can improve the conductivity of layers. Previous results show that the photovoltaic performances can be improved due to the mixed spacers, cations, and anions and decreasing the defect state concentration. Large cations in the perovskite improve the J–V hysteresis^37,38^. The addition of Cs ion to the perovskite film enhances *V*_*OC*_, according to the difference in the perovskite valence band and HTM (i.e., CIS) conduction band energy. This results in the quasi-Fermi level separation at the interface of perovskite/TiO_2_, which provides higher *V*_*OC*_ by increasing the difference in energy levels^28^. As known, the perovskites based on the MA/FA mixture are defect-prone and hence show instability in the presence of humidity^39^. However, accompanied Cs^+^ can reduce lattice defects, which improves the *J*_*SC*_ by decreasing the charge recombination rate^39^.

**Figure 4:**
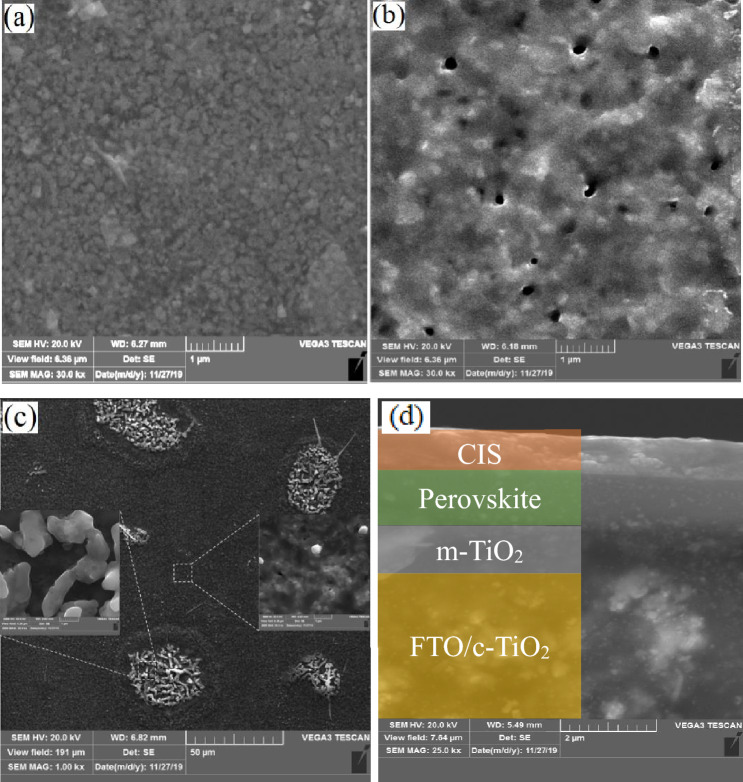
FESEM image of the PSCs surface for (**a**) reference, (**b**) heated, (**c**) humidified PSCs, and (**d**) SEM cross-sectional images of a PSC.

To investigate the changes in morphology, we performed a FESEM analysis. Figure 4 exhibits the FESEM image of the PSCs after humidifying, heating, and cross-sectional images of PSC. As shown, heating the cell creates some pinholes in the structure and weakens the layer interfaces. However, by humidifying the cell, some islands are observed at the surface, showing perovskite segregation. Ion segregation to the surface decreases the defects and weak connection at the interfaces and improves the electrical conduction. As a result, one expects an improvement in cell performance after humidifying and heating.

**Figure 5:**
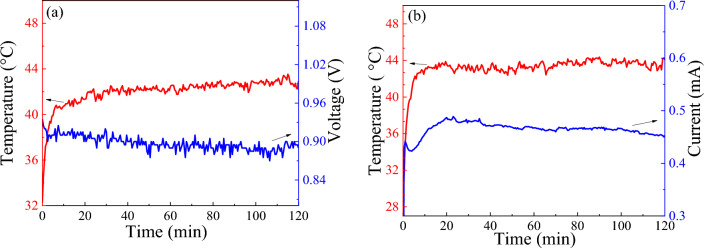
Stability of (**a**) V_OC_ and (**b**) J_SC_ for PSCs in working condition under solar simulator.

The heating stability test of the prepared PSCs points out that the *V*_*OC*_ and *I*_*SC*_ dropped by around 18% and 10% which indicates the good stability of the introduced perovskite. However, stability tests at different days show no considerable changes in cell performance. Figure 5a and b show the stability of *V*_*OC*_ and *J*_*SC*_ by heating under a solar simulator. As shown, heating the PSCs to 45 °C has no considerable effect on cell performance, and a slight decrease in *V*_*OC*_ and *J*_*SC*_ is due to increasing the *R*_*CT*_ and *R*_*s*_. These results suggest that the triple-cation PSC is well-stable under sunlight, which heats the cell typically up to 45 °C.

### Theory and model

The PSCs performance of the device is described by the equivalent circuit model of the *J–V* curve. The equivalent circuit model illustrates the *J–V* curve by:

3 $$ J = \frac{{V - JR_{s} }}{{R_{sh} }} + J_{n} + J_{r} - J_{ph} $$

where $$J_{r} = J_{0} \left( {{\text{exp }}\left( {\frac{qV}{{k_{B} T}}} \right) - 1} \right)$$ and $$J_{n}$$ are the current in the radiative and non-radiative recombination, respectively.$$ J_{ph}$$ is the generated photocurrent by the light that fades out by $$J_{r}$$, $$J_{n}$$, and the Shunt current. $$R_{sh}$$ and $$R_{s}$$ are also the Shunt and the series resistance, respectively.

In the previous work, we modified the standard dynamic electrical model and pointed out the trap-level distribution of charges at the bulk and interfaces^40^. The non-radiative recombination in a solar cell is dominated process through trap density that is defined as follows^39,40^:

4 $$ R = \frac{{np - n_{i}^{2} }}{{\tau_{h} \left( {n + n_{i} \exp \left( {\frac{{E_{t} - E_{i} }}{{k_{B} T}}} \right)} \right) + \tau_{e} \left( {p + n_{i} {\text{exp}}\left( {\frac{{E_{i} - E_{t} }}{{k_{B} T}}} \right)} \right)}} $$

where $\tau_{h}$ and $\tau_{e}$ are electron and hole lifetime, respectively. In addition,* n*_*i*_, *p*, and *n* are the intrinsic density, the densities of holes and electrons, respectively. By inserting *p* and *n* in Eq. (4), the non-radiative recombination becomes:

5 $$ R = \frac{{n_{i} \exp \left( {\frac{{E_{FC} - E_{FV} }}{{k_{B} T}} - 1} \right)}}{{\tau_{h} \left( {{\text{exp}}\left( {\frac{{E_{t} - E_{i} }}{{k_{B} T}}} \right) + \exp \left( { - \frac{{E_{i} - E_{FC} }}{{k_{B} T}}} \right)} \right) + \tau_{e} \left( {{\text{exp}}\left( {\frac{{E_{i} - E_{t} }}{{k_{B} T}}} \right) + {\text{exp}}\left( { - \frac{{E_{FV} - E_{i} }}{{k_{B} T}}} \right)} \right)}} $$

**Figure 6:**
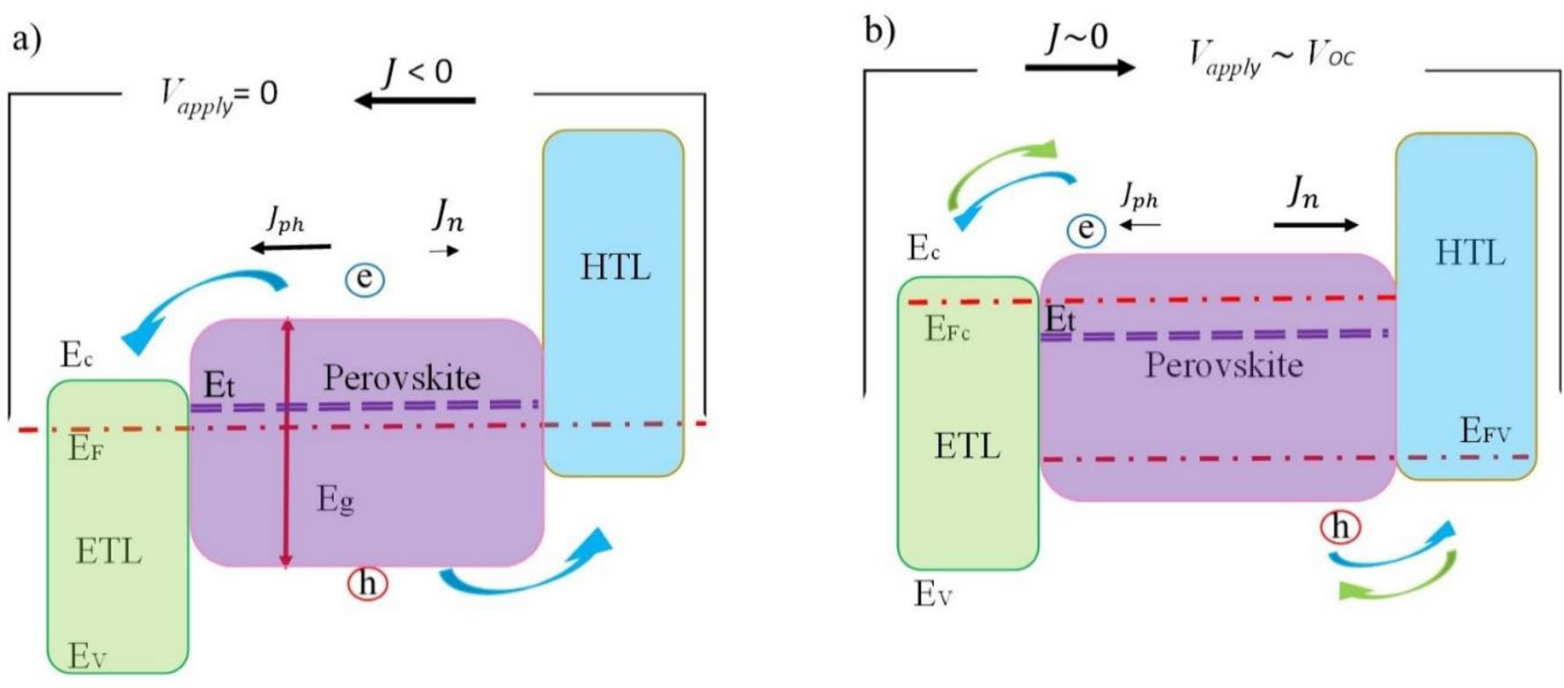
Energy diagram of PSCs to show the behavior of J–V, (**a**) under light illumination (J = J_SC_), (**b**) after voltage bias on the cell. The red and purple dashed lines show Fermi energy and trap levels, respectively.

We show the level energy diagram of the quasi-conduction and quasi-valance level splitting, intrinsic-Fermi energy (*E*_*i*_), and the trap level (${E}_{t}$), in Fig. 6. It schematically shows the different levels before and after applying an external voltage. We take electron and hole lifetime equal that denoted by $\tau$ (i.e., $\tau_{h} \approx \tau_{e} \approx \tau )$^40–42^.

**Figure 7:**
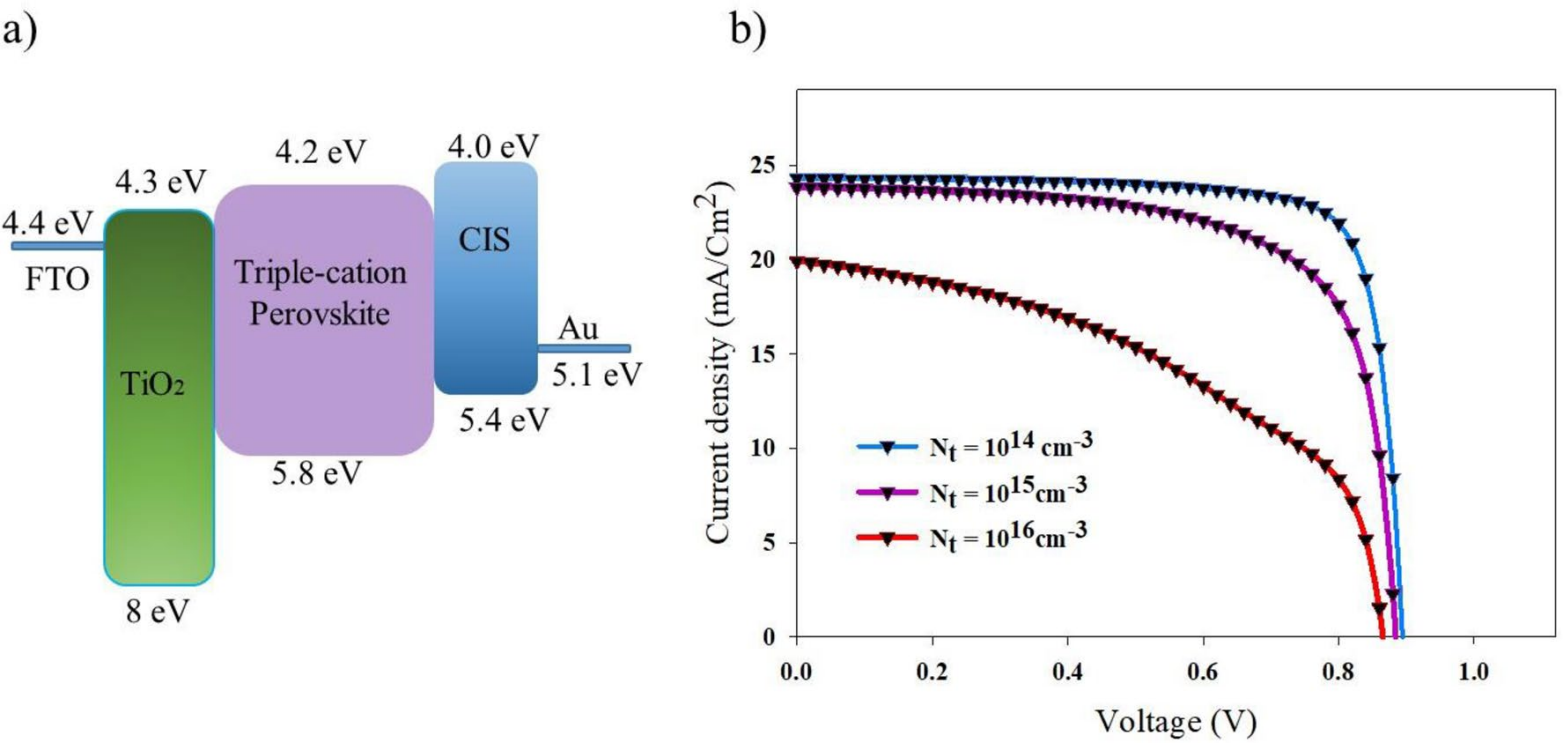
(**a**) Energy level diagram of the PSC with CIS. (**b**) The simulated J–V curves regarding the effect of trap-density values of N_t_ = 10^14^–10^16^ cm^−3^.

Most of the loss of V_OC_ and fill factor (FF) is due to the defect-assisted recombination. The fewer traps-defects led to higher FF and V_OC_ that improve the efficiency of cell. We have studied the dependency of non-radiative recombination on the trap-density and cell performance by Solar Cell Capacitance Simulator (SCAPS) software. SCAPS solves the drift–diffusion equations based on effective parameters involving bandgap energy, donor or acceptor concentration, affinity to illustrate the J–V curve. The studied perovskite solar cell includes: CIS as a p-type layer, TiO_2_ as an n-type layer, and a triple cation perovskite layer as an intrinsic layer (Fig. 7a). Figure 7b shows the performance of the simulated cell by investigating the effect of trap-density values of *N*_*t*_ = 10^14^–10^16^ cm^−3^ in the perovskite layer.

## Conclusions

In this paper, we studied the performance of triple-cation perovskite solar cells after exposure to humidity and heat. FESEM images show that heat creates some pinholes at the surface while humidity causes ion segregation to the surface through pinholes. XRD results and EIS analysis show that the crystallinity of PbI_2_ destroys in humidity and charge transfer resistance decreases accordingly to 1.67 Ω. However, triple-cation PSC is stable and reversible after humidifying and heating with improved performance. Humidified PSCs show *V*_*OC*_ and *J*_*SC*_ of 940 mV and 22.85 mA cm^−2^ with PCE of 14.34%. The increment is due to improving the crystal quality and decreasing the defect state concentration in the perovskite layer. Besides, ion segregation to the PSC layers’ interfaces creates an asymmetric electric field that modifies the cell performance according to the electric field at the interfaces. Results show that working temperature on PSC performances has a negligible effect on acceptable stability and reversibility of *V*_*OC*_ and *J*_*SC*_ after heating up to 45 °C.

## Data Availability

The data that support the findings of this study are available from the corresponding author upon reasonable request.
